# Compatibility of a Silicone Impression/Adhesive System to FDM-Printed Tray Materials—A Laboratory Peel-off Study

**DOI:** 10.3390/ma11101905

**Published:** 2018-10-07

**Authors:** Yichen Xu, Alexey Unkovskiy, Felix Klaue, Frank Rupp, Juergen Geis-Gerstorfer, Sebastian Spintzyk

**Affiliations:** 1 Section Medical Materials Science & Technology, University Hospital Tuebingen, 72076 Tuebingen, Germany; xuyichen10@126.com (Y.X.); felix.klaue@gmx.de (F.K.); frank.rupp@med.uni-tuebingen.de (F.R.); juergen.geis-gerstorfer@med.uni-tuebingen.de (J.G.-G.); 2 Department of Prosthodontics at the Centre of Dentistry, Oral Medicine, and Maxillofacial Surgery with Dental School, University Hospital Tuebingen, 72076 Tuebingen, Germany; dr.unkovskiy@gmail.com; 3 Department of Dental Surgery, Sechenov First Moscow State Medical University, Bolshaya Pirogovskaya Street, 19с1, Moscow 119146, Russia

**Keywords:** additive manufacturing, FDM, custom tray, peel-off strength, surface topography

## Abstract

Computer-aided design (CAD) and additive manufacturing (AM) have shown promise in facilitating the fabrication of custom trays. Due to the clinical requirements, custom tray materials should achieve good bonding to the impression/adhesive systems. This study evaluated the retention of three fused deposition modeling (FDM) custom tray materials to a silicone impression/adhesive system before and after gritblasting (GB) by peel-off test. CAD-designed experimental test blocks were printed by FDM using acrylonitrile butadiene styrene (ABS), polyethylene terephthalate glycol copolyester (PETG), and high impact polystyrene (HIPS), and the reference test blocks were made of a conventional light-curing resin (n = 11). Before and after GB, the surface topography of all tray materials was analysed, and the maximum strength of the test block peeled off from a silicone impression/adhesive system was measured. After GB, the arithmetic mean height (Sa) and the valley fluid retention index (Svi) of the four material groups declined (*p* < 0.05). The peel-off strength of each of the four material groups significantly decreased by GB (*p* < 0.05), but no statistical difference could be found among them before or after GB. In all peel-off tests, adhesive failure occurred at the adhesive-impression material interface. The results indicated ABS, HIPS, and PETG could provide sufficient adhesion to the adhesive as the conventional light-curing resin, and GB could reduce the roughness generated by FDM and weaken the bonding between the adhesive and the silicone impression.

## 1. Introduction

The success of edentulous patients rehabilitation with complete denture prostheses depends on the accuracy of the functional impression [1]. Using well-adapted custom trays can provide a uniform thickness of impression and improve accuracy. However, in conventional techniques, the fabricating process of a custom tray is complicated, time-, and labor-consuming. As digital technologies are emerging in the dental field, computer-aided design (CAD) and additive manufacturing (AM) are expected to simplify this complicated manufacturing process [2].

AM is a promising technology which utilizes CAD to realize the rapid manufacture of an object by means of selective solidification in the horizontal direction and gradual accumulation along the vertical axis [3]. With the benefits of customization and optimization, AM has shown bright prospects of facilitating medical applications in many aspects [4]. Currently, fused deposition modeling (FDM) is one of the most widely used AM technologies [5]. With the advantages of rapid turnaround time, low production costs, and acceptable manufacturing accuracy [6,7,8], FDM technology seems promising for custom tray fabrication. Moreover, recent low-cost FDM 3D-printers are easily affordable for most dental clinics and hospitals. Through a heated nozzle, the FDM 3D-printer can melt and extrude thermoplastic materials, depositing them on a building platform layer by layer, thus manufacturing a model from the bottom up to the top [9]. For the custom trays fabricated by FDM, it is crucial to achieve a good bonding with impression/adhesive systems, particularly when the set impression is being withdrawn from the oral tissues, since even a small detachment of the impression can result in the deformation of the subsequent master cast and the failure of the prosthetic rehabilitation [10,11].

Factors that influence bond strength between custom trays and impression/adhesive systems have been well investigated in previous studies, including custom tray materials [12], impression materials [13], adhesive types [14], adhesive drying time, and surface treatments [15]. Among them, custom tray materials and surface treatments are two critical factors. Dixon et al. found there was a significant difference in the bond strength of a silicone impression/adhesive system to three custom tray materials [16]. Ashwini et al. indicated the vinyl polysiloxane system had a better bond strength to the visible light cure resin than to the autopolymerizing resin [17]. Maruo et al. reported the surface roughness of the tray material significantly affected bond strength [15]. Payne et al. concluded adhesion of the impression/adhesive system to custom tray materials varied with the tray material type and surface topography [18].

However, there is no relevant study focusing on the bonding behavior between FDM-fabricated custom trays and impression/adhesive systems. Therefore, the present study investigated the bonding from the aspects of FDM-printed tray material and surface treatment. At present, a wide variety of thermoplastics are available for FDM technology. These materials vary in their mechanical, physical, and chemical properties. For custom trays, the ideal material should be rigid enough to avoid permanent distortion during impression taking, dimensionally stable in short term before pouring the gypsum, and resistant to temperature and moisture change to prevent shrinkage [19]. Thus, three rigid and dimensionally stable polymers were investigated in this study and compared to a conventional light-curing resin, that is, acrylonitrile butadiene styrene (ABS), polyethylene terephthalate glycol copolyester (PETG), and high impact polystyrene (HIPS). A typical surface treatment technique, gritblasting (GB), was selected for investigation, since it is widely used in dentistry for surface roughening to increase the effective surface area for bonding [20].

Most of the previous studies utilized tensile strength to investigate bonding between the impression/adhesive systems and the custom trays [14,21,22]. However, during impression taking, the detachment between the impression and dental arch always begins either on the front or back side and is never simultaneous. Thus, using peel-off strength to investigate bonding seems to be more appropriate in terms of clinical practice.

The present study aimed to investigate the retention of three selected FDM-printed tray materials (ABS, PETG, and HIPS) to a clinically applied silicone impression/adhesive system through a peel-off test. The effects of the tray material type and GB on the surface roughness and the peel-off bond strength were assessed. Additionally, we sought for 3D-roughness measures which might be related to interfacial peel-off strength. In addition to surface height measures, such as average roughness (Sa) or skewness (Ssk), we checked functional volume measures which are related to the fluid retention property.

## 2. Materials and Methods

### 2.1. CAD of Test Blocks

**Figure 1:**
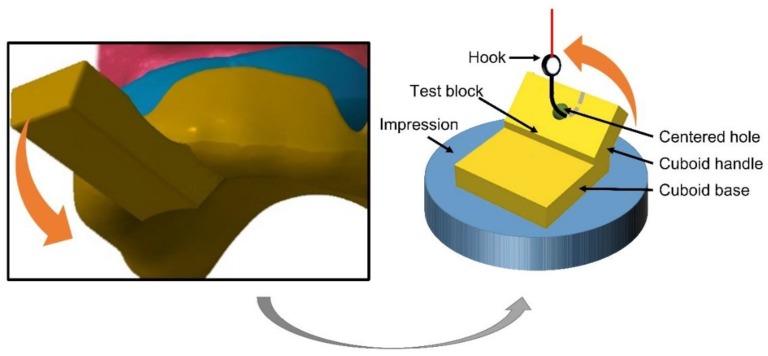
After impression setting, withdrawal is usually achieved by adding a prying force onto the handle of the custom tray. In this process, the impression has the possibility to be peeled off from the tray if they do not have enough bond strength. The test block was used to simulate the peel-off action of the custom tray and measure the maximum peel-off strength.

In order to measure peel-off strength between tray material and a silicone impression material, a test block was designed in a CAD software (OpenSCAD, 2015.03-2 Windows, http://www.openscad.org/). The geometrical shape of the test block was modified from that in published literature [23] to simulate the peel-off action of the custom tray: A cuboid base was designed with a dimension of 25.4 mm × 25.4 mm × 6 mm, and a cuboid handle was added onto its top at an angle of 45 degrees with a centered hole (Figure 1).

### 2.2. AM of Test Blocks

**Figure 2:**
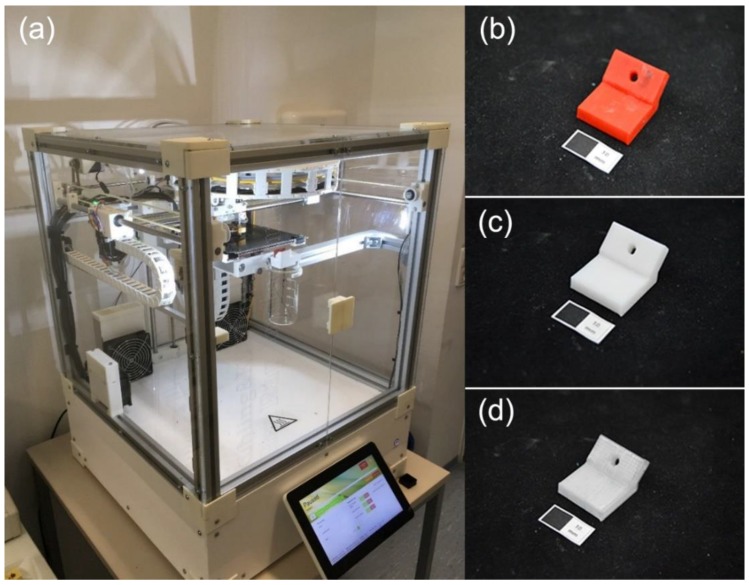
Additive manufacturing of test blocks. (**a**) RepRap Industrial fused deposition modeling (FDM) 3D-printer; (**b**) Test block printed with ABS; (**c**) Test block printed with HIPS; (**d**) Test block printed with PETG (white scale: 10 mm).

The Standard Tessellation Language (STL) file of the modeled test block was sent to an FDM 3D-printer (RepRap Industrial, Kühling&Kühling, Kiel, Germany). Test blocks (n = 11 per group, 33 in total) were manufactured from ABS, HIPS, and PETG, respectively (Figure 2). The 3D-printing setting of each material is shown in Table 1.

### 2.3. Fabrication of Reference Test Blocks

**Figure 3:**
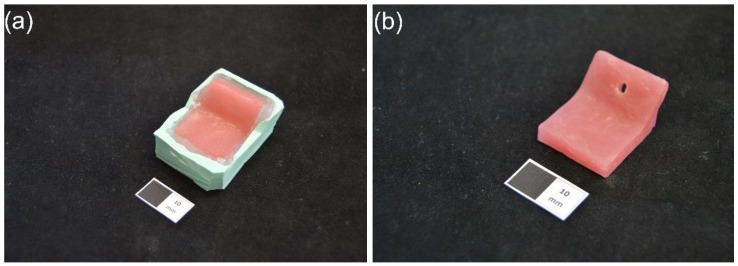
Fabrication of reference test blocks. (**a**) The silicone impression (green) and light-curing resin (red), (**b**) Reference test block (white scale: 10 mm).

The reference test blocks were made of a light-curing resin (Lichtwachs, Wegold Edelmetalle, Wendelstein, Germany), which is commonly used for fabricating custom trays in the conventional technique. To duplicate the 3D-printed test blocks using the light-curing resin, firstly, a silicone impression (Zetalabor Platinum 85, Zhermack, Marl am Dümmer, Germany) was taken from the 3D-printed test block. Then, the light-curing resin was adapted layer by layer into the impression. According to normal dental laboratory procedure, a UV-light device (LML2000, Wilde, Walluf, Germany) was used to cure the first layer for 3 min. Another UV-light device (Visio Beta vario, 3M ESPE, Seefeld, Germany) was used to cure the subsequent layers for 4 min and another 4 min in vacuum to finally fabricate the reference test blocks (Figure 3).

### 2.4. Roughness Measurement

Before and after GB (see Section 2.6), three samples of each material were investigated by a profilometer (S6P, Mahr, Goettingen, Germany) equipped with a needle (90°, 2 μm). The tested area was on the bottom surface of each block, covering 3 mm × 3 mm with 121 profiles included. After roughness measurement, the data of the profilometer were imported into roughness analysis software (MountainsMap Universal 7.3, Digital Surf, Besancon, France). The original data were filtered through a Gaussian Filter (ISO 16610-61) and the cut-off value was set at 0.6 mm. Finally, the 3D views of all surfaces were reconstructed to visualize the effects of different materials and GB on topographies. Two surface height measures, including arithmetic mean height (Sa) and Skewness (Ssk) (ISO 25178-2), and two functional volume measures, that is, core fluid retention index (Sci) and valley fluid retention index (Svi) [24], were calculated to disclose possible correlations between specific surface roughness measures and the observed peel-off strength.

### 2.5. Peel-off Strength Test

**Figure 4:**
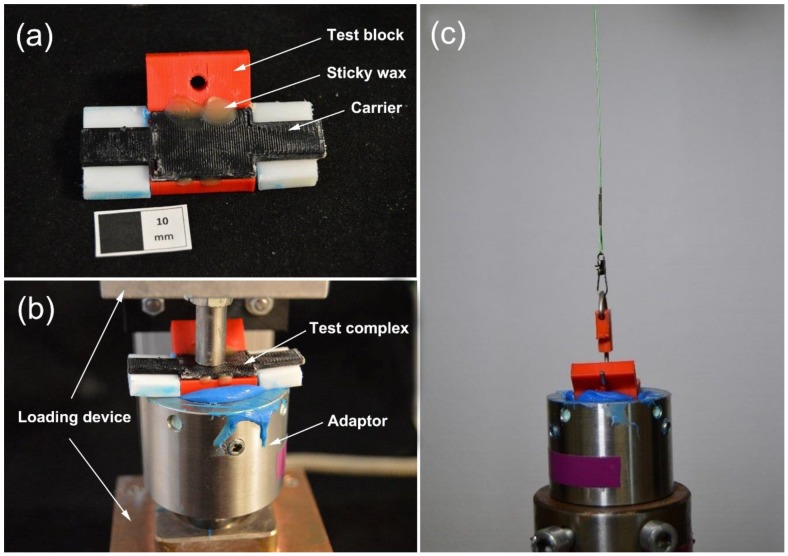
Peel-off strength test. (**a**) A carrier was glued to an acrylonitrile butadiene styrene (ABS) test block using sticky wax to make a test complex (white scale: 10 mm); (**b**) Before impression setting, the test complex was fixed on the cylindrical adaptor by a custom-made loading device with a perpendicular weight of 1.4 kg; (**c**) Peel-off test on the universal testing machine.

Samples of each group were cleaned by a steam jet cleaner (D-S 100 A, Harnisch + Rieth, Winterbach, Germany) and dried in air. After that, a carrier was glued to each test block by molten sticky wax (Supradent–Wachs, Oppermann, Bonn, Germany) to make a test complex (Figure 4a). According to the manufacturer’s instructions, the adhesive (sili fluid, DETAX, Ettlingen, Germany) was smeared on the test surface (bottom of the test block) two times and dried in air for 1 min. Silicone impression material (Xantopren L blue, Heraeus Kulzer, Hanau, Germany) and activator (Activator universal Plus, Heraeus Kulzer, Hanau, Germany) were uniformly mixed at a ratio of 1:1 and then transferred into a cylindrical adaptor. Before silicone setting, the test complex was fixed onto the adaptor by a custom-made loading device [25] with a perpendicular weight of 1.4 kg to simulate the compression force applied to the custom tray during impression taking (Figure 4b). Since the carrier contacted with the adaptor border, a reproducible impression depth of the test block was ensured when subjected to the vertical load, thereby standardizing the impression thickness to 6 mm. The subsequent impression setting time was 3 min for each sample. After setting, the carrier was removed and the excess silicone around the test block was detached. The adaptor was fixed in a universal testing machine (Z010, Zwick, Ulm, Germany). A tension force was applied to the test block through a hook with a crosshead speed of 300 mm/min (Figure 4c). The distance and force were recorded until the test block was peeled off. After peeling, all test surfaces were inspected microscopically (M400, Wild, Heerbrugg, Switzerland). To calculate the area of the test surface, the width of each test surface was measured by a digital caliper (DIGI-MET, PREISSER, Gammertingen, Germany). The peel-off strength σ (N/m) was calculated by Equation (1), where W represents the width (m) and F represents the maximum peel-off force (N) of each test block.

σ = F/W(1)

### 2.6. GB

The test surface of each sample was gritblasted by 125 μm alumina (Cobra, Renfert, Hilzingen, Germany) in a GB device (P-G 400, Harnisch + Rieth, Winterbach, Germany) for 20 s. The alumina particles were propelled by compressed air with a pressure of 0.2 MPa. For standardization, the nozzle of the GB device, which was controlled by a metal holder, was perpendicular to the test surface with a distance of 10 mm. After GB, the samples were cleaned by a steam jet cleaner (D-S 100 A, Harnisch + Rieth, Winterbach, Germany) and dried in air.

Roughness and peel-off strength of all gritblasted samples were measured again, as described above.

### 2.7. Scanning Electron Microscopy (SEM)

In order to specifically analyze the surface topography of the samples before and after GB, test samples (n = 1 per group) were sputtered with a 20 nm thick Au–Pd coating (SCD 050, Baltec, Lübeck, Germany) and then observed in SEM (Leo 1430, Zeiss, Jena, Germany) with 200× magnification at 10 kV acceleration voltage.

### 2.8. Statistical Analysis

The data of peel-off strength were checked for normal distribution within each group. A Scheirer–Ray–Hare test was performed to investigate the effects of GB and the material type on peel-off strength (R 3.5.1, R Foundation for Statistical Computing, Vienna, Austria). The effects of GB and the material type on Sa, Ssk, Sci, and Svi were analyzed by two-way analysis of variance (ANOVA) (SPSS 20.0, IBM, New York, NY, USA). The critical value was set to 0.05.

## 3. Results

### 3.1. 3D Surface Reconstruction

**Figure 5:**
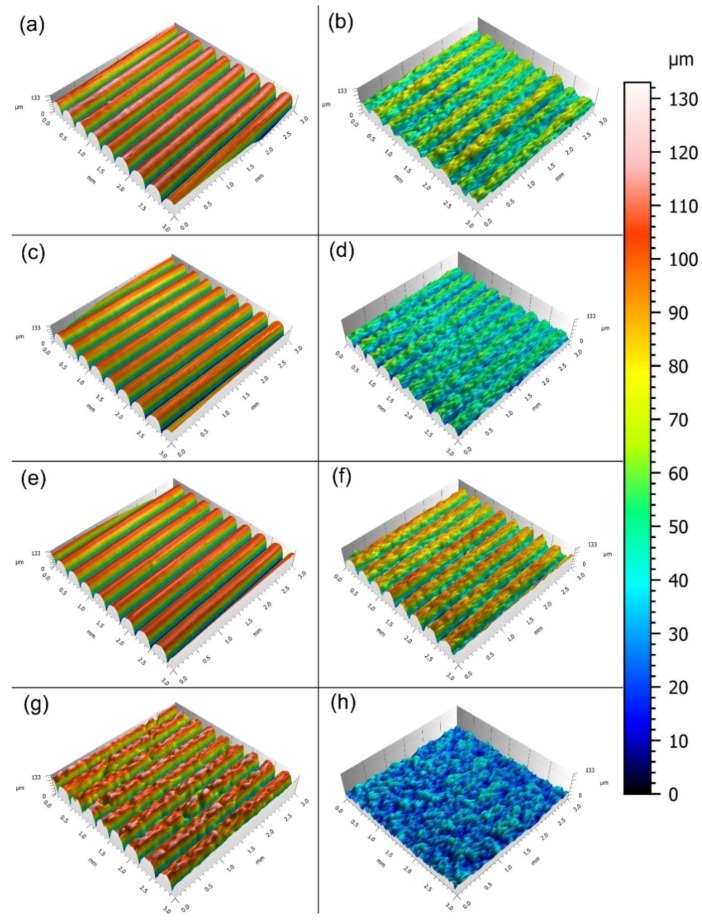
Reconstructed 3D surface topographies of the test blocks. (**a**) ABS before gritblasting (GB); (**b**) ABS after GB; (**c**) high impact polystyrene (HIPS) before GB; (**d**) HIPS after GB; (**e**) polyethylene terephthalate glycol copolyester (PETG) before GB; (**f**) PETG after GB; (**g**) Reference resin before GB; (**h**) Reference resin after GB.

As shown in Figure 5, before GB, the surfaces of ABS, HIPS, PETG, and reference resin blocks (Figure 5a,c,e,g respectively) had clearly discernible waveforms. After GB, the respective waveforms on the surfaces of each group were subject to varying degrees of erosion. Among them, the erosion on the surfaces of the PETG group (Figure 5f) was the lowest (surface height shown in red and orange), but the waveforms could still be identified. Followed by ABS and HIPS (Figure 5b,d), the waveforms became blurred and difficult to identify. The surfaces of the reference group (Figure 5h) experienced the most severe erosion (surface height shown in blue), where the waveforms completely disappeared.

### 3.2. SEM

**Figure 6:**
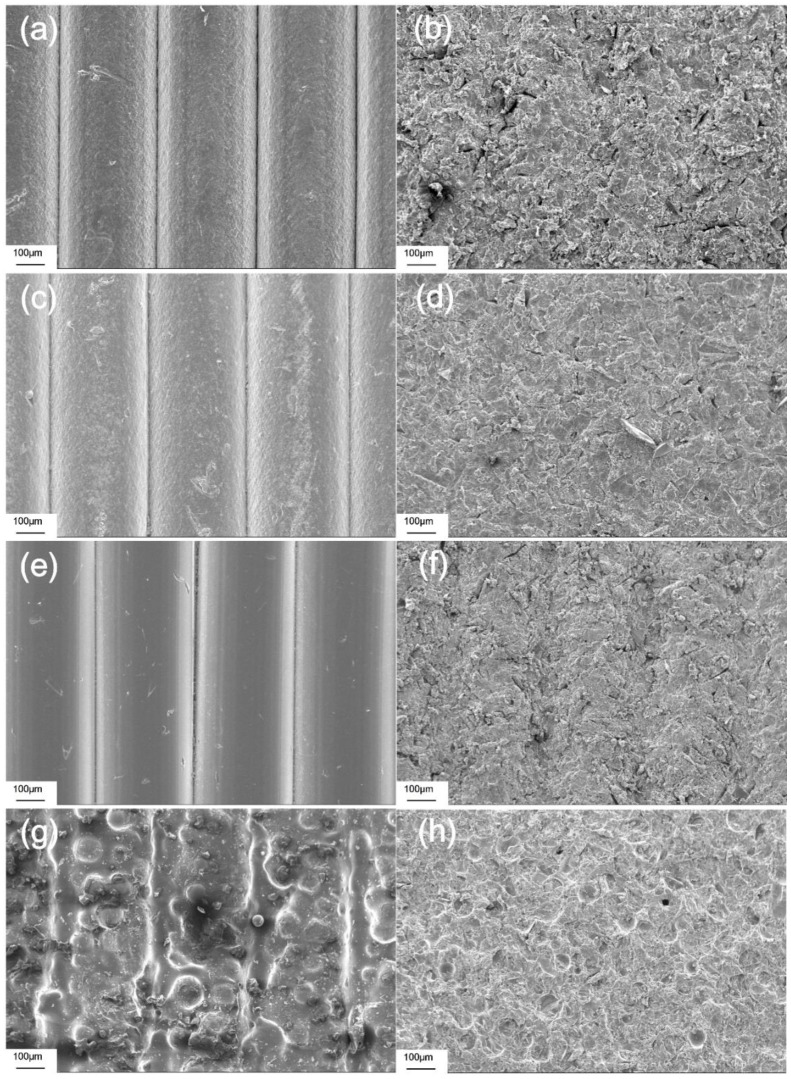
SEM pictures with 200× magnification. (**a**) ABS before GB; (**b**) ABS after GB; (**c**) HIPS before GB; (**d**) HIPS after GB; (**e**) PETG before GB; (**f**) PETG after GB; (**g**) Reference resin before GB; (**h**) Reference resin after GB.

The SEM pictures in Figure 6 show the more detailed surface topography of each material group. Before GB, the peaks and valleys of the waveforms could be clearly identified in all material groups. The surfaces of the FDM-printed test blocks had almost no microroughness structure (Figure 6a,c,e), whereas the surface of the reference test block formed apparent microroughness structures (Figure 6g). After GB, the material of each group was eroded from their surfaces in different degrees, and peaks and valleys became unclear (Figure 6b,d,f) or completely disappeared (Figure 6h). After GB, microroughness structures could be found on the surfaces of all material groups.

### 3.3. Roughness Measurements

**Figure 7:**
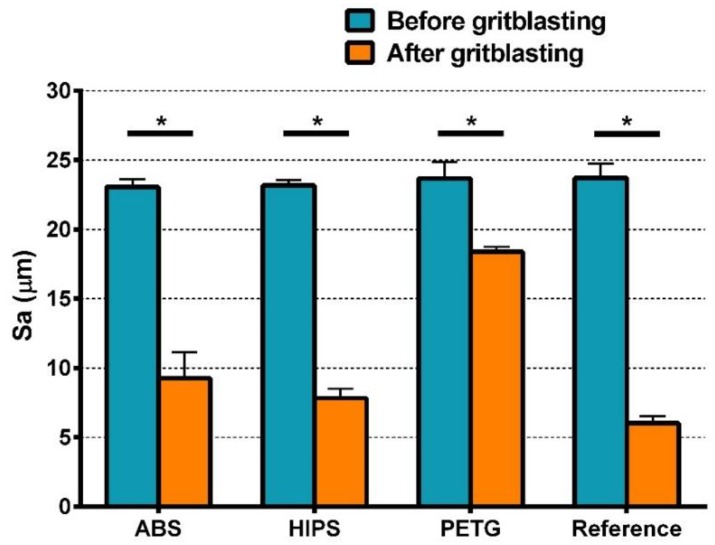
Sa values of all material groups before and after GB (Mean ± SD, n = 3). * represents statistically significant differences in Sa of all groups before and after GB.

The results of two-way ANOVA of Sa indicate that the type of material, GB, and their interactions had significant effects on the Sa value (Table S1). The change in Sa shows a similar trend as the result of 3D surface reconstruction (Figure 7): Before GB, there was no statistical difference among the four groups. After GB, the Sa value of each group was significantly lower than that before GB. As shown in Table 2, the Sa value of the gritblasted PETG group was significantly higher than that of the other three gritblasted groups, followed by the ABS and HIPS groups, which did not significantly differ. After GB, the reference group had the lowest Sa value, which differed statistically from the ABS group, but not from the HIPS group.

**Figure 8:**
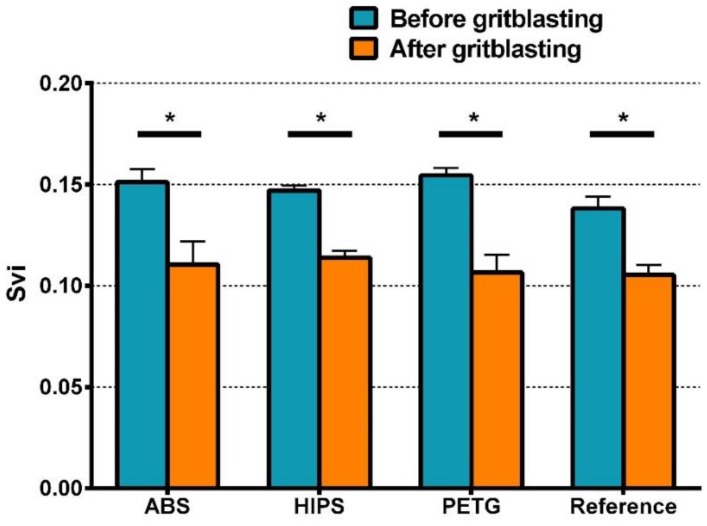
Svi values of all material groups before and after GB (Mean ± SD, n = 3). * represents statistically significant differences in Svi of all groups before and after GB.

However, two-way ANOVA of Svi shows different results (Table S2): Only GB demonstrated significant effects on Svi, whereas the type of material and their interactions had no significant effects. As Figure 8 indicates, after GB, the Svi value of each group became lower, but no statistical difference could be found among the four gritblasted groups. As shown in Table 3, before GB, slight statistical differences could be found between the PETG and reference group, but the mean Svi values of all four groups were very close to each other.

### 3.4. Peel-off Strength Test

**Figure 9:**
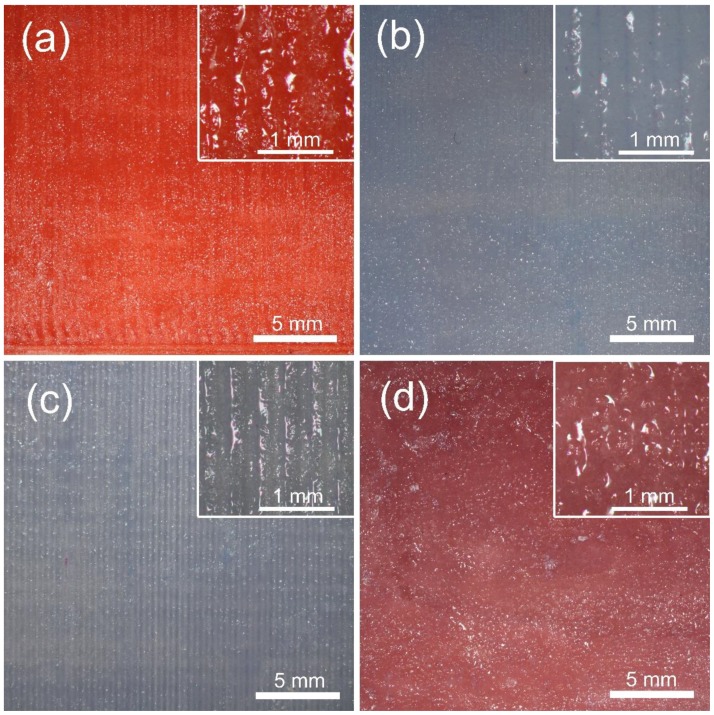
Light microscopical pictures of test surfaces after peeling (6.3× and 32× magnification). (**a**) ABS; (**b**) HIPS; (**c**) PETG; (**d**) Reference resin.

As shown in Figure 9, the adhesive remained completely on all peeled test surfaces, and no impression material residues could be found.

**Figure 10:**
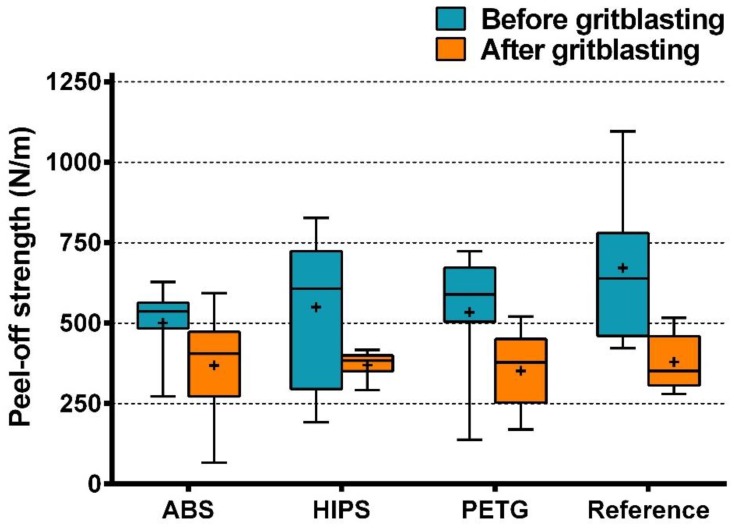
Peel-off strength of all material groups before and after GB (Boxplot, n = 11). + represents the mean peel-off strength of each group.

The results of the normality tests are shown in Table S3. Statistical analysis reveals GB significantly affected peel-off strength, but the type of material and their interactions had no significant effect (Table S4). As shown in Figure 10, before and after GB, there was no statistical difference in peel-off strength among the four material groups. After GB, the mean peel-off strength of each group was lower than that before GB.

Before and after GB, the data of peel-off strength showed a pattern similar to that of Svi, but not to that of Sa. As for Sa, the results of Ssk and Sci did not show any pattern related to the data of peel-off strength.

## 4. Discussion

Conventionally, the materials for custom tray fabrication mainly include autopolymerizing resins, thermoplastic resins, and light-curing resins. Among them, light-curing resins are regarded as the gold standard, since they are dimensionally stable, stiff, and can be used immediately after setting [19]. The light-curing resin was also reported to have a better bond strength to the impression/adhesive systems than the autopolymerizing resin [12,16,17,26]. Recently, FDM has shown promise in facilitating the fabrication of custom trays in dentistry, but only a few studies are related to this topic. Zhi et al. reported the accuracy of the custom trays made by FDM was comparable to that of hand-made custom trays [27], and Hu et al. found out that the accuracy of the custom trays made by FDM was even better [28]. In addition, a recent publication indicated that making custom trays through FDM was more efficient with less manufacturing time [29]. However, research on the bonding between FDM-fabricated custom trays and impression/adhesive systems is still lacking.

Elastomeric impression materials are widely used in dentistry. According to the chemical composition, they are categorized as polysulfide, condensation silicone, addition silicone, and polyether. Each category is further divided into different viscosities. These different elastomeric impression materials with different viscosities vary in wettability, flexibility, and tear strength, which may affect bonding to custom trays. In addition, Payne et al. and Maruo et al. indicated that different impression materials required specific surface topographies to achieve optimum bond strength [15,18]. In this study, a light-body condensation silicone impression material, used clinically for manually fabricating custom trays, was investigated. Other impression materials with different viscosities could be investigated in further studies.

**Figure 11:**
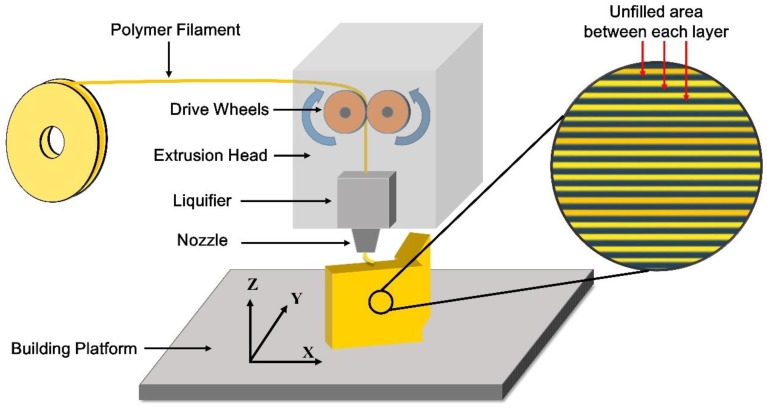
The working principle of the FDM technique. During the layered manufacturing process of FDM, the unfilled area can be found between each deposited layer, resulting in the original roughness on the FDM-printed surface.

GB utilizes high-speed stream to abrade the surface of objects, which is a common surface processing method in dentistry and usually used to roughen the surface before bonding [30,31]. However, it is worth noting that if a surface is rough enough, GB will smooth it. As shown by the results of reconstructed 3D surface topographies (Figure 5) and roughness measures (Figure 7 and Figure 8), the surfaces of all test blocks became smoother after GB, signifying that the surfaces manufactured by FDM are rough already. This can be related to the working principle of the FDM technique (Figure 11): A numerically controlled nozzle moves in X- and Y-axis across the building platform, extruding thermoplastic materials to generate a 2D layer with a specific thickness. Successive 2D layers melt together in Z-axis to build up a 3D object. In this process, the unfilled area can be found between each 2D layer, thus making the original waveforms on the printed surface [32]. The orientation of the texture on the printed surface is perpendicular to the Z-axis (build direction). As Figure 11 indicates, this study aimed to investigate the factors of tray material and surface treatment, so the texture orientations of all test blocks were standardized to one direction. However, the effects of texture orientations cannot be ignored and may affect peel-off strength, so further studies should be performed to evaluate the orientation effects, not only on the test blocks, but also on the original custom trays. Generally, the roughness generated by FDM is deemed the most significant drawback of this technique [33]. For certain end-use in engineering applications, it usually needs post-processing techniques to eliminate the manufactured roughness [34]. However, to improve the retention of impression materials, the roughness as generated by FDM seems beneficial, and the GB in dentistry cannot achieve such rough topographies.

As Figure 6 indicates, the SEM pictures show the surface topographies with higher magnification. Since the reference resin test blocks were chemically fabricated, microroughness structures could be found on the surface (Figure 6g). By contrast, the 3D-printed test blocks were fabricated by physical melting and solidification, and few microroughness structures formed (Figure 6a,c,e). As shown in Figure 6b,d,f, GB decreased surface roughness by reducing the vertical distance between the peaks and valleys, thus making them difficult to identify. However, at the same time, many irregular microroughness structures were generated. The decrease in roughness and the increase in microroughness finally had a negative effect on peel-off strength (Figure 10), indicating that, in this study, compared to microroughness, roughness may act as the main factor that affects peel-off bond strength.

In the present study, a stylus profilometer was used to investigate surface roughness. Since the stylus could not fully enter every small fissure or pore on the surfaces, the resolution in Z-axis was limited by the radius of the stylus tip. Using optical methods, for example, white light interferometry, could provide more accurate data for further studies.

After GB, the Sa values of all materials decreased in different degrees and the Sa value of PETG was significantly higher than the Sa of the other three materials. Sa is an amplitude measure, which describes the mean height of a sampling area [35]. Therefore, after GB, the waveforms on the PETG surface were preserved best, signifying the surface of PETG experienced the least erosion and showed the best abrasion resistance. This has been confirmed by 3D topographies and SEM (Figure 5 and Figure 6). However, the wear behavior of polymers is complicated and the abrasion resistance of polymers is related to a number of mechanical parameters in different models, with test results also differing depending upon the test method and polymer type [36].

**Figure 12:**
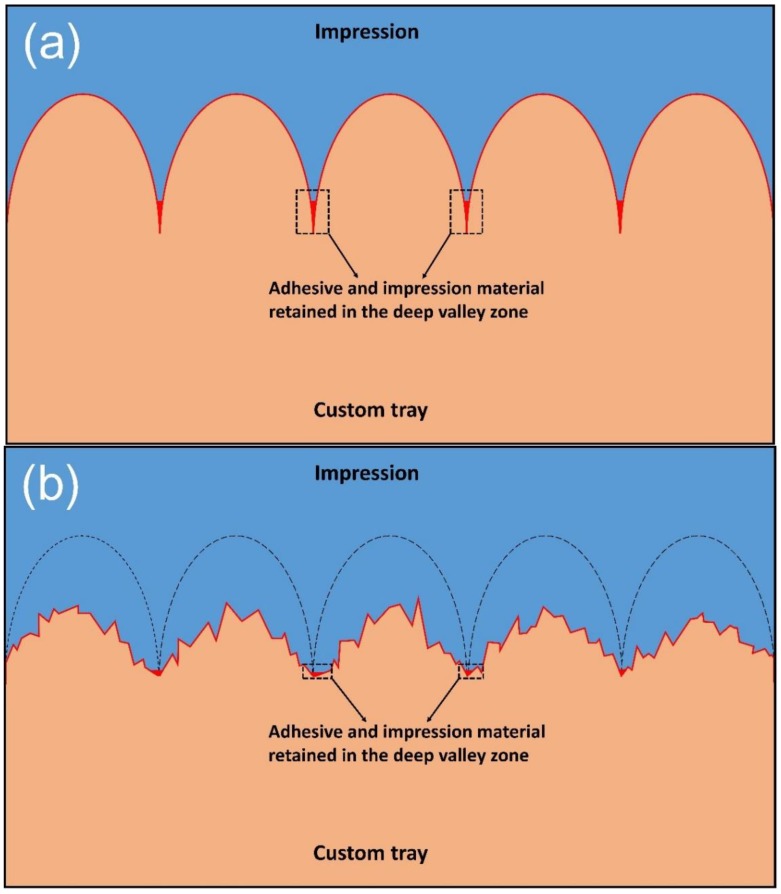
The adhesive and impression material retained in the deep valley zone. (**a**) Before GB; (**b**) After GB.

The roughness measure Svi shows a different behavior, that is, the Svi values of all groups declined after GB, but no significant difference could be found among them. Svi is an index of fluid retention in the valley zone [24]. A larger value of Svi indicates better fluid retention in the deepest valleys of the surface. For a Gaussian surface, the value of Svi is approximately 0.11, and a Svi value larger than 0.11 represents the dominance of void volume in the valley zone [37]. Through GB, the Svi value of the four materials decreased from about 0.15 to about 0.11 (Figure 8), indicating a certain loss of void volume, and the fluid retention capacity declined. Therefore, after GB, less adhesive and impression material were retained by the deepest valley zones, which, after setting, provided crucial mechanical interlocking for bond strength (Figure 12).

In studies of bonding between custom trays and impression/adhesive systems, different crosshead speeds have been utilized, ranging from 5 mm/min to 508 mm/min [16,23,38,39]. Since the strain rate may influence the result of bond strength, the crosshead speed should be related to the application of the material. In this study, a crosshead speed of 300 mm/min was chosen, which was thought to be approximate to the speed at which the dentist withdraws the custom tray out of patients’ oral cavity and to closely simulate clinical practice.

Since impression materials do not usually have any chemical adhesion with the tray materials [40,41], tray adhesives are commonly applied before impression taking to guarantee a reliable adhesion. In a bonding system consisting of impression material, adhesive, and tray material, any rupture might cause bonding failure. Different modes of adhesive failure have been reported in previous studies, and ruptures may occur at two weak sites: At adhesive-impression material and at adhesive-tray material interfaces [13]. Since each type of impression has its compatible adhesive, the modes of rupture may vary in different tray materials. If the tray material cannot achieve a bonding to the adhesive stronger than the impression material does, rupture may occur at the adhesive-tray material interface, and bond strength might be influenced by the tray material type. Conversely, if the tray material has enough bond strength to the adhesive, the rupture may happen at the adhesive-impression material interface. In this case, the tray material type may have no effect on bond strength. In this study, the applied adhesive remained completely on the peeled surfaces, and no impression material residues could be found. This indicates that the ruptures occurred at the adhesive-impression material interface and good adhesion was achieved between the four investigated tray materials and the adhesive for the condensation silicone.

Peel-off strength is defined as the average load per unit width of the bonded sample. However, in previous studies, the areal stress was utilized for investigating the peel bond strength between the custom tray and the impression/adhesive system [23,42]. Since during the peeling process, the peel force is always applied on the bonding line, it was thought that using the line peel stress for investigation is more appropriate. Therefore, the data of peel bond strength in previous studies were recalculated for comparison. MacSween et al. reported there were significant differences in peel-off strength among different impression/adhesive systems, and the mean peel-off strength values ranged from 4606 N/m to 21324.8 N/m [42]. In this study, the mean peel-off strength of the condensation silicone was 500.3 to 671.3 N/m before GB and 368.3 to 379.4 N/m after GB, which exceeds that reported by Grant et al. (269.8 to 315.4 N/m for condensation silicone) [23]. The possible reason for the observed higher peel-off strength might be the different experimental methods applied and the increased roughness of the test surface. More importantly, the mode of adhesive failure was completely different. In the study of Grant et al., adhesive failure occurred at the adhesive-tray material interface, indicating the investigated tray material (acrylic resin) did not achieve sufficient adhesion to the adhesive. As a result, the adhesive-tray material interface was weaker than the adhesive-impression material interface.

In order to investigate the effect of the material type on peel-off strength, the light-curing resin test blocks were the replicas of 3D-printed ones. Thus, all the test blocks were manufactured to have, as much as possible, the same surface topography before GB. The results of statistical analysis indicate the four material types did not differ in their peel-off strength. As mentioned above, this is because the four investigated tray materials achieved sufficient adhesion to the adhesive, and the peel-off strength in this study derived from the bonding between the impression and the adhesive. However, this result does not indicate the FDM-fabricated custom trays and the conventional light-curing custom trays have the same bond strength with the impression. In the fabricating process of conventional custom trays, the light-curing resin is adapted over the smooth wax spacer [9], so the actual roughness and peel-off strength of light-curing custom trays may be lower than that found in the present study. Moreover, wax residue will further affect bonding and reduce peel-off strength [24]. Therefore, compared to conventional custom trays made of light-curing resins, the FDM-printed ones may have a better bonding to the impression material. The results of the Scheirer–Ray–Hare test demonstrate that GB significantly decreased peel-off strength, signifying that surface roughness will contribute to the bonding between the adhesive and the impression. It seems unreasonable to explain peel-off strength based on Sa, since, after GB, statistical differences could be found in the Sa values of the four material groups and the Sa value of PETG was significantly higher, but the peel-off strength of the four materials had no significant difference. Similarly, in addition to the most widely used 3D roughness measure Sa, the roughness measures Ssk and Sci investigated in the present study could not be related to peel-off strength. By contrast, the results of Svi and peel-off strength showed a certain degree of consistency. Therefore, it seems reasonable to suggest that the adhesive and impression material retained in the deepest valley zones of the tray materials’ topography may play an important role in the bonding between the adhesive and the impression.

As this study was designed with the use of experimental test blocks in the laboratory, future studies should further confirm the clinical function of FDM-printed custom trays in an oral cavity environment. Moreover, further analysis investigating other bonding factors, such as impression materials and adhesive types, is required.

## 5. Conclusions

1. The three investigated FDM-printed tray materials (ABS, HIPS, and PETG) could provide sufficient adhesion to the adhesive compared to the conventional light-curing resin.
2. Custom trays made by FDM seems to possess intrinsic surface roughness that might improve bonding.
3. GB could reduce the surface roughness of FDM-printed custom tray materials and thus weaken the bonding between the adhesive and the impression.
4. The Svi index of the tray materials’ topography may have a certain correlation with the peel-off strength between the adhesive and the impression.

To sum up, FDM could be a good alternative to the conventional procedure of manufacturing custom trays, especially when a digital workflow is applied.

## Figures and Tables

**Table 1:** 3D printing settings of each material.

Settings	ABS	HIPS	PETG
Printing bed temperature	105 °C	110 °C	60 °C
Printing chamber temperature	70 °C	70 °C	0–35 °C
Printing nozzle temperature	275 °C	235 °C	275–285 °C
Printing speed	28 mm/s	28 mm/s	28 mm/s
Layer thickness	0.35 mm	0.35 mm	0.35 mm
Infill	100%	100%	100%

**Table 2:** Mean (standard deviation) Sa value of each group after GB. The results of statistical analysis were shown by superscript letters. The groups with the same superscript letter had no statistical difference.

Group after GB	Sa (μm)
ABS	9.26 (1.86) ^A^
HIPS	7.82 (0.68) ^AC^
PETG	18.40 (0.35) ^B^
Reference	6.03 (0.50) ^C^

**Table 3:** Mean (standard deviation) Svi value of each group before GB. The results of statistical analysis were shown by superscript letters. The groups with the same superscript letter had no statistical difference.

Group before GB	Svi
ABS	0.151 (0.007) ^AB^
HIPS	0.147 (0.003) ^AB^
PETG	0.155 (0.004) ^A^
Reference	0.138 (0.006) ^B^

## Supplementary Materials

The following are available online at http://www.mdpi.com/1996-1944/11/10/1905/s1, Table S1: Two-way ANOVA of Sa, Table S2: Two-way ANOVA of Svi, Table S3: Normality test of peel-off strength, Table S4: Scheirer-Ray-Hare test of line peel-off strength.

## References

[1] Petrie C.S., Walker M.P., Williams K. (2005). A survey of US prosthodontists and dental schools on the current materials and methods for final impressions for complete denture prosthodontics. J. Prosthodont..

[2] Schmitt S., Schmitt R. Going Digital: Unlimited Potential for Dentures. http://schmittdds.com/PDF/GoingDigital_Unlimited_potential_for_dentures.pdf.

[3] Gebhardt A. (2016). 3D-Drucken: Grundlagen und Anwendungen des Additive Manufacturing (AM).

[4] Zanetti E.M., Aldieri A., Terzini M., Calì M., Franceschini G., Bignardi C. (2017). Additively manufactured custom load-bearing implantable devices: Grounds for caution. Australas. Med. J..

[5] Stansbury J.W., Idacavage M.J. (2016). 3D printing with polymers: Challenges among expanding options and opportunities. Dent. Mater..

[6] Huang S.H., Liu P., Mokasdar A., Hou L. (2013). Additive manufacturing and its societal impact: A literature review. Int. J. Adv. Manuf. Technol..

[7] Maschio F., Pandya M., Olszewski R. (2016). Experimental Validation of Plastic Mandible Models Produced by a “Low-Cost” 3-Dimensional Fused Deposition Modeling Printer. Med. Sci. Monit..

[8] Unkovskiy A., Spintzyk S., Axmann D., Engel E.-M., Weber H., Huettig F. (2017). Additive manufacturing: A comparative analysis of dimensional accuracy and skin texture reproduction of auricular prostheses replicas. J. Prosthodont..

[9] Marro A., Bandukwala T., Mak W. (2016). Three-Dimensional Printing and Medical Imaging: A Review of the Methods and Applications. Curr. Probl. Diagn. Radiol..

[10] Saunders W.P., Sharkey S.W., Smith G.M., Taylor W.G. (1991). Effect of impression tray design and impression technique upon the accuracy of stone casts produced from a putty-wash polyvinyl siloxane impression material. J. Dent..

[11] Terry D.A., Tric O., Blatz M., Burgess J.O. (2010). The custom impression tray: Fabrication and utilization. Dent. Today.

[12] Dixon D.L., Breeding L.C., Bosser M.J., Nafso A.J. (1993). The effect of custom tray material type and surface treatment on the tensile bond strength of an impression material/adhesive system. Int. J. Prosthodont..

[13] Chai J.Y., Jameson L.M., Moser J.B., Hesby R.A. (1991). Adhesive properties of several impression material systems: Part I. J. Prosthet. Dent..

[14] Kumar S., Gandhi U.V., Banerjee S. (2014). An In Vitro Study of the Bond Strength of Five Adhesives Used for Vinyl Polysiloxane Impression Materials and Tray Materials. J. Indian Prosthodont. Soc..

[15] Maruo Y., Nishigawa G., Oka M., Minagi S., Irie M., Suzuki K. (2007). Tensile bond strength between custom tray and elastomeric impression material. Dent. Mater. J..

[16] Dixon D.L., Breeding L.C., Brown J.S. (1994). The effect of custom tray material type and adhesive drying time on the tensile bond strength of an impression material/adhesive system. Int. J. Prosthodont..

[17] Ashwini B.L., Manjunath S., Mathew K.X. (2014). The Bond Strength of Different Tray Adhesives on Vinyl Polysiloxane to Two Tray Materials: An In Vitro Study. J. Indian Prosthodont. Soc..

[18] Payne J.A., Pereira B.P. (1995). Bond strength of two nonaqueous elastomeric impression materials bonded to two thermoplastic resin tray materials. J. Prosthet. Dent..

[19] Thongthammachat S., Moore B.K., Barco M.T., Hovijitra S., Brown D.T., Andres C.J. (2002). Dimensional accuracy of dental casts: Influence of tray material, impression material, and time. J. Prosthodont..

[20] Byeon M.S., Lee H.M., Bae S.T. (2017). Shear Bond Strength of Al2O3 Sandblasted Y-TZP Ceramic to the Orthodontic Metal Bracket. Materials.

[21] Peregrina A., Land M.F., Wandling C., Johnston W.M. (2005). The effect of different adhesives on vinyl polysiloxane bond strength to two tray materials. J. Prosthet. Dent..

[22] Yi M.-H., Shim J.-S., Lee K.-W., Chung M.-K. (2009). Drying time of tray adhesive for adequate tensile bond strength between polyvinylsiloxane impression and tray resin material. J. Adv. Prosthodont..

[23] Grant B.E., Tjan A.H.L. (1988). Tensile and peel bond strengths of tray adhesives. J. Prosthet. Dent..

[24] Stout K.J., Blunt L. (2000). Three Dimensional Surface Topograpgy.

[25] Sawada T., Spintzyk S., Schille C., Schweizer E., Scheideler L., Geis-Gerstorfer J. (2016). Influence of Different Framework Designs on the Fracture Properties of Ceria-Stabilized Tetragonal Zirconia/Alumina-Based All-Ceramic Crowns. Materials.

[26] Abdullah M.A., Talic Y.F. (2003). The effect of custom tray material type and fabrication technique on tensile bond strength of impression material adhesive systems. J. Oral Rehabil..

[27] Huang Z., Wang X., Hou Y. (2015). Novel Method of Fabricating Individual Trays for Maxillectomy Patients by Computer-Aided Design and Rapid Prototyping. J. Prosthodont..

[28] Chen H., Yang X., Chen L., Wang Y., Sun Y. (2016). Application of FDM three-dimensional printing technology in the digital manufacture of custom edentulous mandible trays. Sci. Rep..

[29] Wei L., Chen H., Zhou Y.S., Sun Y.C., Pan S.X. (2017). Evaluation of production and clinical working time of computer-aided design/computer-aided manufacturing (CAD/CAM) custom trays for complete denture. J. Peking Univ. Heal. Sci..

[30] Grewal Bach G.K., Torrealba Y., Lagravère M.O. (2013). Orthodontic bonding to porcelain: A systematic review. Angle Orthod..

[31] Mair L., Padipatvuthikul P. (2010). Variables related to materials and preparing for bond strength testing irrespective of the test protocol. Dent. Mater..

[32] Gajdoš I., Slota J. (2013). Influence of printing conditions on structure in FDM prototypes. Teh. Vjesn..

[33] Alsoufi M.S., Elsayed A.E. (2017). How Surface Roughness Performance of Printed Parts Manufactured by Desktop FDM 3D Printer with PLA+ is Influenced by Measuring Direction. Am. J. Mech. Eng..

[34] Ituarte I.F., Chekurov S., Salmi M., Tuomi J., Partanen J. (2015). Post-processing opportunities of professional and consumer grade 3D printing equipment: A comparative study. Int. J. Rapid Manuf..

[35] Kournetas N., Spintzyk S., Schweizer E., Sawada T., Said F., Schmid P., Geis-Gerstorfer J., Eliades G., Rupp F. (2017). Comparative evaluation of topographical data of dental implant surfaces applying optical interferometry and scanning electron microscopy. Dent. Mater..

[36] Shipway P.H., Ngao N.K. (2003). Microscale abrasive wear of polymeric materials. Wear.

[37] Jarnstrom J., Sinervo L., Toivakka M., Peltonen J. (2007). Topography and gloss of precipitated calcium carbonate coating layers on a model substrate. Tappi. J..

[38] Davis G.B., Moser J.B., Brinsden G.I. (1976). The bonding properties of elastomer tray adhesives. J. Prosthet. Dent..

[39] Bindra B. (1997). Adhesion of elastomeric impression materials to trays. J. Oral Rehabil..

[40] Ona M., Takahashi H., Sato M., Igarashi Y., Wakabayashi N. (2010). Effect of reactive adhesives on the tensile bond strength of polyvinyl siloxane impression materials to methyl methacrylate tray material. Dent. Mater. J..

[41] Lakshmi C.B.S., Umamaheswari B., Devarhubli A.R., Pai S., Wadambe T.N. (2018). An evaluation of compatibility of three different impression materials to three different tray acrylic materials using tray adhesives: An In vitro Study. Indian J. Dent. Sci..

[42] MacSween R., Price R.B. (1991). Peel bond strengths of five impression material tray adhesives. J. Can. Dent. Assoc..

