# Supplementary material for: Compatibility of a Silicone Impression/Adhesive System to FDM-Printed Tray Materials—A Laboratory Peel-off Study
Source: Materials (Basel). 2018 Oct 7;11(10):1905. doi: 10.3390/ma11101905 (PMC6213137; doi:10.3390/ma11101905)
Supplement: Supplementary file 1 [file materials-11-01905-s001.pdf]

Table S1. Two-way ANOVA of Sa.

Source	Type III Sum of Squares	df	Mean Square	F	Sig.	Partial Eta Squared
Corrected Model	1291.682[a]	7	184.526	202.119	< 0.001	0.989
gritblasting	1017.513	1	1017.513	1114.523	< 0.001	0.986
materials	142.492	3	47.497	52.026	< 0.001	0.907
gritblasting * materials	131.677	3	43.892	48.077	< 0.001	0.900
Error	14.607	16	0.913			
Total	8151.806	24				
Corrected Total	1306.289	23				

[a]. R Squared = 0.989 (Adjusted R Squared = 0.984)

Table S2. Two-way ANOVA of Svi.

Source	Type III Sum of Squares	df	Mean Square	F	Sig.	Partial Eta Squared
Corrected Model	0.010 [a]	7	0.001	32.139	< 0.001	0.934
gritblasting	0.009	1	0.009	211.296	< 0.001	0.930
materials	0.000	3	1.181E-004	2.776	0.075	0.342
gritblasting * materials	0.000	3	7.586E-005	1.783	0.191	0.251
Error	0.001	16	4.254E-005			
Total	0.406	24				
Corrected Total	0.010	23				

[a]. R Squared = 0.934 (Adjusted R Squared = 0.905)

Table S3. Normality test of peel-off strength.

		Shapiro-Wilk		
		Statistic	df	Sig.
Before gritblasting	ABS	0.768	11	0.004
	HIPS	0.907	11	0.222
	PETG	0.795	11	0.008
	REFERENCE	0.941	11	0.530
After gritblasting	ABS	0.905	11	0.213
	HIPS	0.932	11	0.427
	PETG	0.966	11	0.839
	REFERENCE	0.920	11	0.315

Table S4. Scheirer-Ray-Hare test of line peel-off strength.

	Sum of Squares	df	Mean Square	H	Sig.
Material	1074	3	358	1.645	0.649
Gritblasting	18271	1	18271	27.994	1.217E-07
Material*Gritblasting	1352	3	451	2.072	0.558
Residuals	36085	80	451		
